# Hybrid Interacting Multiple Model Filtering for Improving the Reliability of Radar-Based Forward Collision Warning Systems

**DOI:** 10.3390/s22030875

**Published:** 2022-01-24

**Authors:** Jung Min Pak

**Affiliations:** Department of Electrical Engineering, Wonkwang University, 460 Iksan-Daero, Iksan 54538, Korea; destin11@wku.ac.kr

**Keywords:** automotive radar, finite impulse response filter, forward collision warning, interacting multiple model, probabilistic data association

## Abstract

Automotive forward collision warning (FCW) systems based on radar sensors attracted widespread attention in recent years. To achieve a reliable FCW, it is essential to accurately estimate the position and velocity of a preceding vehicle. To this end, this study proposed a novel estimation algorithm, which is a hybrid of interacting multiple model probabilistic data association (IMM-PDA) and finite impulse response (FIR) filters. Although the IMM-PDA filter is one of the most successful algorithm for tracking a maneuvering target in clutters, it sometimes exhibits divergence owing to modeling errors. In this study, the divergent IMM-PDA filter in the novel algorithm was reset and recovered using an assisting FIR filter. Consequently, this enabled reliable estimation for FCW. The improved reliability of the proposed algorithm was demonstrated through the simulation of preceding vehicle tracking using automotive radars.

## 1. Introduction

In recent years, advanced driver-assistance systems (ADAS) were developed and attracted widespread attention [1]. Among various ADAS, forward collision warning (FCW) systems are essential for the safety of drivers [2]. FCW can be implemented using various sensors, such as radar [3], vision [4], and global positioning system (GPS) [5]. Radar-based FCW is the most commonly used FCW owing to the fact that radars can provide long-range detection regardless of weather/light conditions [6,7,8].

Studies on FCW algorithms focused on the decision-making process involved in determining whether to alert a collision. In typical FCW systems [9,10,11,12], the time-to-collision (TTC) is computed using the information of the relative distance and velocity between the host and preceding vehicles, and a warning is provided if the TTC was below a certain threshold. Recent studies [13,14,15] analyzed the behaviors (driving patterns) of vehicles using artificial intelligence (AI) algorithms, and a warning is provided in situations with a high risk of collision. Although various decision-making algorithms for FCW were developed, most of them are based on information on the state (position and velocity) of preceding vehicles. Thus, it is essential to develop algorithms that can accurately estimate the state of a preceding vehicle to achieve a reliable FCW [5,9,11].

The estimation of the position and velocity of a target, known as target tracking, requires two processes: signal processing and data processing. In radar signal processing, measurements of the range, bearing, and range rate are obtained by processing the received radar signals, and this can be achieved using various signal processing methods including Fourier analysis, filtering, and AI algorithms. The measurements obtained using signal processing contain noises and clutters; hence, a postprocessing, known as data processing, is required. Stochastic filters (e.g., Kalman filter (KF)) are used in data processing to improve the accuracy of the estimation of the position and velocity of a target. In addition, data association algorithms (e.g., probabilistic data association) are used to address the problem of clutters (i.e., false measurements). Probabilistic data association filter (PDAF) [16], which is the combination of KF and probabilistic data association, is one of the most renowned and successful algorithms for single target tracking in the presence of clutters. Particularly, interacting multiple model PDAF (IMMPDAF) [17,18,19,20] is an advanced algorithm for maneuvering targets, and joint PDAF [21] is an algorithm for multiple target tracking. Recently, various multiple target tracking algorithms, such as multiple hypothesis tracking [22] and probability hypothesis density filter [23], were developed. This study focused on data processing, particularly, the problem of single vehicle tracking in the presence of clutters, for application in FCW systems. Thus, IMMPDAF was used as the main filter in the proposed algorithm.

However, as the IMMPDAF is based on the KF, it sometimes exhibits estimation divergence owing to modeling errors. For example, a significant difference in the actual movement of a target and the movement predicted by the motion model may result in the divergence of KF. This divergence could be attributed to the accumulation of errors in the KF. To overcome the filter divergence problem, stochastic filters with finite impulse response (FIR) structures were investigated and developed [24,25,26,27,28,29,30,31]. FIR stochastic filters, known as FIR filters, only utilize recent finite measurements to produce a current state estimate, which makes them robust against modeling errors. However, because they utilize finite measurements, the accuracy of their estimates are generally lower than that of KF that utilizes all past measurements. In summary, the IMMPDAF is accurate under normal conditions, but may diverge if the actual movement of a target is significantly different from that of the motion model. In contrast, FIR filters do not diverge, but are less accurate under normal conditions.

Therefore, this study proposed a novel hybrid estimation algorithm for improving the reliability of radar-based FCW. The proposed algorithm utilized IMMPDAF as the main filter, and an FIR filter as an assisting filter. The assisting FIR filter only function when the IMMPDAF diverges, and it produces a state estimate and covariance. In this situation, the IMMPDAF is reset using the information obtained from the FIR filter. Consequently, the IMMPDAF recovers from failures. Therefore, the proposed algorithm was named hybrid IMM-PDA/FIR filter (HIPFF). The performance of the proposed HIPFF was demonstrated using simulations, and the relative position and velocity of a vehicle were estimated using the HIPFF and conventional IMMPDAF. The results revealed that when a preceding vehicle decelerated rapidly, the conventional IMMPDAF often failed and diverged, whereas the proposed HIPFF did not. This indicates that HIPFF significantly improved the reliability of the state estimation for FCW systems.

The rest of this paper is organized as follows. Section 2 introduces the problem involved in the estimation of the position and velocity of a preceding vehicle in the presence of radar clutters. Section 3 introduces the IMMPDAF algorithm, and Section 4 introduces the proposed HIPFF algorithm. Section 5 presents the simulation results to demonstrate the performance of the proposed HIPFF, and the conclusions are drawn in Section 6.

## 2. Preceding Vehicle State Estimation Using Automotive Radars

**Figure 1:**
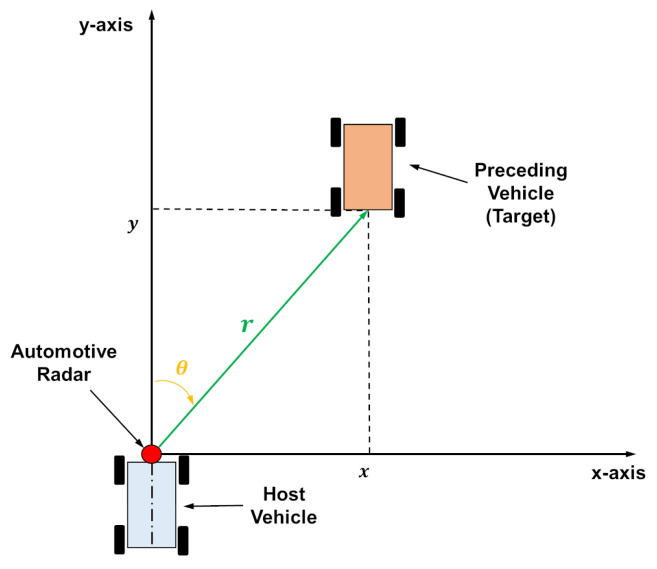
Automotive radar geometry.

This section describes the problem involved in the estimation of the state of a preceding vehicle using an automotive radar in the presence of clutters. The state (position and velocity) of the preceding vehicle is represented using a two-dimensional (2D) Cartesian coordinate system (Figure 1). An automotive radar for FCW is installed in the middle of the host vehicle, and the origin of the coordinate system is located at the position of the radar. The x-axis is aligned with the longitudinal direction of the host vehicle. The state of a preceding vehicle (target) is composed of 2D positions, (x,y), and velocities, $\left(v_{x},v_{y}\right)$. Consequently, the state vector is defined as $x={\left[x\;v_{x}\;y\;v_{y}\right]}^{T}$.

Estimation algorithms require state-space models, which consist of motion and measurement models. The motion model expresses the transition of the state vector in discrete-time. Typically, the motion model used for target tracking is the discrete white noise acceleration (DWNA) model, which can be represented using:

(1) $$\begin{array}{cc} x(k+1) & =Fx(k)+Gw(k),\\ F & =\left[\begin{array}{cccc} 1 & T & 0 & 0\\ 0 & 1 & 0 & 0\\ 0 & 0 & 1 & T\\ 0 & 0 & 0 & 1 \end{array}\right],\;\;G=\left[\begin{array}{cc} T^{2}/2 & 0\\ T & 0\\ 0 & T^{2}/2\\ 0 & T \end{array}\right], \end{array}$$

where x(k) is the state vector at a discrete time *k* and w(k) is the zero-mean white Gaussian process noise vector. The covariance of w(k) is defined as $Q\left(k\right)=\sigma_{w}^{2}\;I_{2}$, where $\sigma_{w}^{2}$ is the variance of process noise, and $I_{2}$ is the 2×2 identity matrix.

The process noise, w(k), corresponds to the acceleration of a target, and $\sigma_{w}^{2}$ determines the level of acceleration. This indicates that $\sigma_{w}^{2}$ reflects the amount of changes in velocity. When an appropriate value of $\sigma_{w}^{2}$ is used, the filter can produce accurate state estimates. However, if $\sigma_{w}^{2}$ is not sufficiently large, and there is a significant change in the velocity of a target, the filter divergence phenomenon (i.e., rapid increase in estimation error) may occur. In contrast, if $\sigma_{w}^{2}$ was sufficiently large and the changes in velocity was too small, the estimation accuracy is degraded. Thus, a mismatch between the model (i.e., $\sigma_{w}^{2}$) and the actual motion of a target can result in a degradation in the estimation accuracy or even filter divergence. However, the acceleration of a target is generally unknown, and $\sigma_{w}^{2}$ is determined using a rule of thumb based on the knowledge of engineers. The IMM estimation algorithm can solve the problem of determining the uncertain $\sigma_{w}^{2}$. The IMM algorithm utilizes multiple models that use different $\sigma_{w}^{2}$. A detailed algorithm for the IMM estimation is described in Section 3.

The radar reports the relative range, *r*, and bearing, θ, between the host and preceding vehicle at a constant time interval, *T*, as shown in Figure 1. These radar measurements including noise can be expressed as

(2) $$\begin{array}{cc} r_{m} & =r+{\bar{v}}_{r},\\ \theta_{m} & =\theta +{\bar{v}}_{\theta }, \end{array}$$

where *r* and θ are the true range and bearing, respectively; $r_{m}$ and $\theta_{m}$ are the measured range and bearing, respectively; and ${\bar{v}}_{r}$ and ${\bar{v}}_{\theta }$ are the measurement noises, which are assumed to be mutually independent and zero-mean white Gaussian. The variances of ${\bar{v}}_{r}$ and ${\bar{v}}_{\theta }$ are denoted as $\sigma_{r}^{2}$ and $\sigma_{\theta }^{2}$, respectively.

The measurement model relates the radar measurements with the state, x(k). However, the raw radar measurements are polar coordinates, and they should be converted to Cartesian coordinates. The conversion can be written as

(3) $$\begin{array}{cc} x_{m} & =r_{m}\;cos\left(\theta_{m}\right),\\ y_{m} & =r_{m}\;sin\left(\theta_{m}\right), \end{array}$$

where $x_{m}$ and $y_{m}$ are the converted measurements, and they can also be represented as

(4) $$\begin{array}{cc} x_{m} & =x+{\bar{v}}_{x},\\ y_{m} & =y+{\bar{v}}_{y}, \end{array}$$

where ${\bar{v}}_{x}$ and ${\bar{v}}_{y}$ are the measurement noises in the Cartesian coordinate.

The measurement vector is defined as $z\left(k\right)={\left[x_{m}\left(k\right)\;y_{m}\left(k\right)\right]}^{T}$, and the measurement model is represented as follows:

(5) $$\begin{array}{cc} z(k) & =Hx\left(k\right)+\bar{v}\left(k\right),\\ H & =\left[\begin{array}{cccc} 1 & 0 & 0 & 0\\ 0 & 0 & 1 & 0 \end{array}\right], \end{array}$$

where $\bar{v}\left(k\right)$ is the measurement noise vector. The noise covariance of converted measurement, denoted as R(k), is obtained as follows [17]:

(6) $$\begin{array}{cc} R(k) & =\left[\begin{array}{cc} R_{11}\left(k\right) & R_{12}\left(k\right)\\ R_{12}\left(k\right) & R_{22}\left(k\right) \end{array}\right],\\ R_{11}\left(k\right) & =r_{m}{\left(k\right)}^{2}\;\sigma_{\theta }^{2}\;sin^{2}\left(\theta_{m}\left(k\right)\right)+\sigma_{r}^{2}\;cos^{2}\left(\theta_{m}\left(k\right)\right),\\ R_{22}\left(k\right) & =r_{m}{\left(k\right)}^{2}\;\sigma_{\theta }^{2}\;cos^{2}\left(\theta_{m}\left(k\right)\right)+\sigma_{r}^{2}\;sin^{2}\left(\theta_{m}\left(k\right)\right),\\ R_{12}\left(k\right) & =\left(\sigma_{r}^{2}-r_{m}{\left(k\right)}^{2}\;\sigma_{\theta }^{2}\right)\;sin\left(\theta_{m}\left(k\right)\right)\;cos\left(\theta_{m}\left(k\right)\right). \end{array}$$

Given the motion and measurement models, Equations (1) and (5), the state x(k) can be estimated using various estimation algorithms. However, two factors hinder this estimation: radar clutters and maneuvering of target. Radar clutters are false measurements generated by the scattering of radar reflection. Because it is unknown whether a measurement is true or false, false measurements make the estimation difficult. Nevertheless, this uncertainty in measurements can be handled by PDA algorithm. Maneuvering corresponds to changes in the direction of movement and speed. A rapid and significant maneuvering of a target results in a mismatch between the motion model (particularly $\sigma_{w}^{2}$) and the actual motion of the target, which can result in divergence of the estimation algorithm. This uncertainty in the motion model can be handled by IMM algorithm. In the following section, IMMPDAF, which is a combination of IMM and PDA algorithms, is introduced.

## 3. Interacting Multiple Model (IMM) Probabilistic Data Association (PDA) Filter

The IMMPDAF operates multiple PDA filters in parallel, and each PDA filter is matched to a model. The inputs and outputs of the multiple PDA filters were mixed using the IMM algorithm. In this section, first, the PDA filter is introduced, after which the IMM algorithm is described.

The PDA filter is a recursive estimator, indicating that the processes are repeatedly performed at each time step. The first process of the PDA filter is a time update process, in which the state estimate and estimation error covariance are updated using the motion model as follows:

(7) $$\begin{array}{cc} \hat{x}\left(k|k-1\right) & =F\hat{x}\left(k-1|k-1\right),\\ P(k|k-1) & =FP\left(k-1|k-1\right)F^{T}+GQ\left(k\right)G^{T}. \end{array}$$

where the notation “k|k−1” corresponds to *a priori* estimates obtained at time *k* using the measurements up to time k−1. In addition, the notation “k−1|k−1” corresponds to *a posteriori* estimates obtained at k−1 using the measurements up to k−1.

The second process is measurement validation, in which only “valid” measurements are selected. The number of measurements obtained at time *k* is denoted as m(k), and *i*-th measurement (i=1,2,⋯,m(m)) at time *k* is denoted as $z_{i}\left(k\right)$. The measurements within the validation region (also known as the gate) are selected as follows:

(8) $$\begin{array}{cc} \nu_{i}\left(k\right) & ={\left(z_{i}\left(k\right)-\hat{z}\left(k\right)\right)}^{T}S_{i}^{-1}\left(k\right)\left(z_{i}\left(k\right)-\hat{z}\left(k\right)\right)\leq \gamma ,\\ \hat{z}\left(k\right) & =H\hat{x}\left(k|k-1\right),\\ S_{i}\left(k\right) & =HP\left(k|k-1\right)H^{T}+R_{i}\left(k\right), \end{array}$$

where γ is the gate threshold, which can be obtained from a *chi-square* table, and $R_{i}\left(k\right)$ is the measurement noise covariance computed using $z_{i}\left(k\right)$ and (6). The number of selected (validated) measurements is denoted as n(k).

The third process is the computation of association probability. The association probability, $\beta_{i}\left(k\right)$, is the probability that the measurement $z_{i}\left(k\right)$ is from the true target (i.e., true measurement), which can be computed as follows:

(9) $$\begin{array}{cc} \beta_{i}\left(k\right) & =\left\{\begin{array}{c} \frac{L_{i}\left(k\right)}{1-P_{D}P_{G}+\sum_{j=1}^{n(k)}L_{j}\left(k\right)}\;\;\;\;i=1,\cdots ,n\left(k\right)\\ \frac{1-P_{D}P_{G}}{1-P_{D}P_{G}+\sum_{j=1}^{n(k)}L_{j}\left(k\right)}\;\;\;\;i=0 \end{array}\right. \end{array}$$

(10) $$\begin{array}{cc} L_{j}\left(k\right) & =\frac{\exp[-0.5{\left(z_{j}\left(k\right)-\hat{z}\left(k\right)\right)}^{T}S_{j}^{-1}\left(k\right)\left(z_{j}\left(k\right)-\hat{z}\left(k\right)\right)]}{{\left(2\pi \right)}^{n_{z}/2}{\left|R_{j}\left(k\right)\right|}^{1/2}\;n\left(k\right)}\;P_{D}\;V_{j}\left(k\right) \end{array}$$

(11) $$\begin{array}{cc} V_{j}\left(k\right) & =c_{n_{z}}\gamma^{n_{z}/2}{\left|S_{j}\left(k\right)\right|}^{1/2}, \end{array}$$

where i=0 corresponds to the case where there is no true measurement, and $V_{j}\left(k\right)$ is the volume of the validation region, which is computed as

(12) $$\begin{array}{cc} V_{k}\left(j\right) & =c_{n_{z}}\gamma^{n_{z}/2}{\left|S_{k}\left(j\right)\right|}^{1/2}, \end{array}$$

where $n_{z}$ is the dimension of the measurement vector. In addition, $P_{G}$ and $P_{D}$ in (9) are the gate probability and the target detection probability, respectively.

The last process is the measurement update, in which state estimates are updated using the validated measurements as follows:

$$\begin{array}{cc} \hat{x}\left(k|k\right) & =\hat{x}\left(k|k-1\right)+\sum_{i=1}^{n(k)}W_{i}\left(k\right)\;\beta_{i}\left(k\right)\;\nu_{i}\left(k\right),\\ W_{i}\left(k\right) & =P\left(k|k-1\right)H^{T}S_{i}^{-1}\left(k\right),\\ \nu_{i}\left(k\right) & =z_{i}\left(k\right)-\hat{z}\left(k\right),\\ P(k|k) & =\bar{P}\left(k|k\right)+\tilde{P}\left(k|k\right),\\ \bar{P}\left(k|k\right) & =\beta_{0}\left(k\right)\;P\left(k|k-1\right)+\sum_{i=1}^{n(k)}\beta_{i}\left(k\right)\left(I-W_{i}\left(k\right)H\right)P\left(k|k-1\right), \end{array}$$

(13) $$\begin{array}{cc} \tilde{P}\left(k|k\right) & =\sum_{i=1}^{n(k)}\beta_{i}\left(k\right)W_{i}\left(k\right)\nu_{i}\left(k\right)\nu_{i}^{T}\left(k\right)W_{i}^{T}\left(k\right)\\ \; & -\left(\sum_{i=1}^{n(k)}\beta_{i}\left(k\right)W_{i}\left(k\right)\nu_{i}\left(k\right)\right)\left(\sum_{i=1}^{n(k)}\beta_{i}\left(k\right)\nu_{i}^{T}\left(k\right)W_{i}^{T}\left(k\right)\right). \end{array}$$

If there is no measurement in the validation region (i.e., n(k)=0), the measurement update process is omitted.

Next, the IMM algorithm is introduced. In this study, the IMM algorithm uses the following models:

- $M_{1}$: DWNA model (1) assuming undetected target (false target)
- $M_{2}$: DWNA model (1) assuming true target with normal motion
- $M_{3}$: DWNA model (1) assuming true target performing maneuvering
- $M_{4}$: DWNA model (1) assuming true target with nearly constant velocity

The difference between $M_{1}$ and $M_{2}$ is the target detection probability, which can be expressed as follows:

(14) $$\begin{array}{cc} P_{D}^{j} & =\left\{\begin{array}{c} 0,\;\;\mathrm{if}\;\;j=1,\\ P_{D},\;\;\mathrm{if}\;\;j=2,3,4. \end{array}\right. \end{array}$$

where *j* is the model (mode) number. The model $M_{1}$ plays a role in detecting a target loss. The differences among $M_{2}$–$M_{4}$ are the process noise variance $\sigma_{w}^{2}$. $M_{3}$ uses the highest $\sigma_{w}^{2}$, and $M_{4}$ uses the lowest $\sigma_{w}^{2}$. A PDA filter that uses model *j* is denoted as filter *j* (j=1,2,3,4).

First, IMM-PDA algorithm computes the mixing probability as

(15) $$\begin{array}{cc} \mu_{h|j}\left(k-1|k-1\right) & =\frac{1}{{\bar{c}}_{j}}p_{hj}\mu_{h}\left(k-1\right),\\ {\bar{c}}_{j} & =\sum_{h=1}^{r}p_{hj}\mu_{h}\left(k-1\right), \end{array}$$

where $\mu_{h|j}\left(k-1|k-1\right)$ is the mixing probability, indicating the weight imposed on the information from filters *h* to *i*.

Using the mixing probabilities, initial estimate and covariance for the filter *j* are obtained as follows:

(16) $$\begin{array}{cc} {\hat{x}}^{0j}\left(k-1|k-1\right) & =\sum_{h=1}^{4}{\hat{x}}^{0j}\left(k-1|k-1\right)\mu_{h|j}\left(k-1|k-1\right), \end{array}$$

(17) $$\begin{array}{cc} P^{0j}\left(k-1|k-1\right) & =\sum_{h=1}^{4}\mu_{i|j}\left(k-1|k-1\right)\{P^{h}\left(k-1|k-1\right)+[{\hat{x}}^{h}\left(k-1|k-1\right)\\ \; & -{\hat{x}}^{0j}\left(k-1|k-1\right)]{\left[{\hat{x}}^{i}\left(k-1|k-1\right)-{\hat{x}}^{0j}\left(k-1|k-1\right)\right]}^{T}\}. \end{array}$$

${\hat{x}}^{0j}\left(k-1|k-1\right)$ and $P^{0j}\left(k-1|k-1\right)$ are fed to the filter *j*, which corresponds to $\hat{x}\left(k-1|k-1\right)$ and P(k−1|k−1) in (7), respectively. Subsequently, PDA filtering (7)–(13) is performed for each model *j*, and ${\hat{x}}^{j}\left(k|k\right)$ and $P^{j}\left(k|k\right)$ are obtained

Next, the likelihoods for ${\hat{x}}^{j}\left(k|k\right)$ are computed as follows:

(18) $$\begin{array}{cc} \Lambda_{j}\left[Z\left(k\right)\right] & =\frac{1-P_{D}^{j}}{V^{n(k)}}+\frac{n\left(k\right)P_{D}^{j}}{V^{n(k)-1}}\sum_{i=1}^{n(k)}N\left[z_{i}\left(k\right);{\hat{z}}^{j}\left(k|k-1\right),S^{j}\left(k\right)\right], \end{array}$$

(19) $$\begin{array}{cc} V & =\pi \gamma |S^{j}{\left(k\right)|}^{1/2}, \end{array}$$

(20) $$\begin{array}{cc} S^{j}\left(k\right) & =HP^{j}\left(k|k\right)H^{T}+R_{i}\left(k\right), \end{array}$$

(21) $$\begin{array}{cc} {\hat{z}}^{j}\left(k|k-1\right) & =H{\hat{x}}^{j}\left(k|k\right), \end{array}$$

where $N[z_{i}\left(k\right);{\hat{z}}^{j}\left(k|k-1\right),S^{j}\left(k\right)]$ corresponds to the Gaussian probability density function with the mean ${\hat{z}}^{j}\left(k|k-1\right)$ and covariance $S^{j}\left(k\right)$; Z(k) is the set of validated measurements defined as $Z\left(k\right)={\left\{z_{i}\left(k\right)\right\}}_{i=1}^{n(k)}$. Using the likelihoods, mode probabilities are updated as

(22) $$\begin{array}{cc} \mu_{j}\left(k\right) & =\frac{1}{c}\Lambda_{j}\left[Z\left(k\right)\right]{\bar{c}}_{j}, \end{array}$$

(23) $$\begin{array}{cc} c & =\sum_{j=1}^{4}\Lambda_{j}\left[Z\left(k\right)\right]{\bar{c}}_{j}. \end{array}$$

Lastly, the ultimate estimate and covariance at time *k* are obtained by combination as follows:

(24) $$\begin{array}{cc} \hat{x}\left(k|k\right) & =\sum_{j=1}^{4}{\hat{x}}^{j}\left(k|k\right)\mu_{j}\left(k\right), \end{array}$$

(25) $$\begin{array}{cc} P(k|k) & =\sum_{j=1}^{4}\mu_{j}\left(k\right)\left\{P^{j}\left(k|k\right)+\left[{\hat{x}}^{j}\left(k|k\right)-\hat{x}\left(k|k\right)\right]{\left[{\hat{x}}^{j}\left(k|k\right)-\hat{x}\left(k|k\right)\right]}^{T}\right\}. \end{array}$$

At the initial time of the IMMPDAF filtering, the mode probabilities were set equally as $\mu_{j}\left(k\right)=0.25$. The transition probabilities from mode *h* to *j*, which is denoted as $p_{hj}$, is an important design parameter that affects the performance of the IMMPDAF algorithm. If the characteristics of the target motion are known, $p_{hj}$ can be designed using the information on the mean sojourn time for each mode. However, if the target motion information is not available, $p_{hj}$ can be set equally.

The IMMPDAF can handle the problem involved in the selection of a value for uncertain $\sigma_{w}^{2}$ in the motion model by adopting multiple models that use different $\sigma_{w}^{2}$ values. However, the IMMPDAF exhibits certain limitations. First, it has a limited number of models because of computational burden. Second, the multiple models are selected based on the knowledge of engineers, and inappropriate models are sometimes selected. In target tracking problems, even IMMPDAF can fail and diverge if the changes in target velocity are significantly different from the $\sigma_{w}^{2}$ value in the motion model (e.g., rapid deceleration of vehicles owing to full breaking). Thus, this study proposed a novel hybrid filtering algorithm that can recover the IMMPDAF from failures. Failure of IMMPDAF under severe conditions and recovering the failed algorithm were demonstrated using simulation results.

## 4. Hybrid IMM-PDA/Finite Impulse Response (FIR) Filter

In this section, the proposed HIPFF algorithm is introduced. In the HIPFF algorithm, the IMMPDAF acts as the main filter, and a FIR filter was used to assist the main filter. This assisting FIR filter operates when the IMMPDAF algorithm fails. Thus, the first problem is to detect the failures of the IMMPDAF. In this study, target loss indicates that the estimated position is far away from the actual target position. If the IMMPDAF loses the target, $M_{1}$ exhibits the highest mode (model) probability among the four models. The mode probability of $M_{1}$ is denoted as $\mu_{1}\left(k\right)$. Thus, a target loss can be detected using $\mu_{1}\left(k\right)$. However, it is too late to operate the assisting FIR filter after the detection of a target loss. If there are no measurements in the validation region (n(k)=0), the IMMPDAF cannot perform the measurement update, which may result in a target loss. Thus, n(k)=0 can be considered as a symptom of failures. The proposed HIPFF algorithm operated the assisting FIR filter when n(k)=0, and obtained the state estimate and covariance for resetting the main filter. A minimum variance FIR filter (MVFF) was adopted for the HIPFF algorithm. The MVFF is a one-step prediction estimator, and it can produce state estimate and covariance at the current time *k* using the measurements on the horizon k−N≤k≤k−1, where *N* is the horizon size of the FIR filter. Moreover, because the MVFF does not use the current measurement, it is suitable for the employed assisting filter when there is no validated measurements. The equations of the MVFF can be written as follows:

(26) $$\begin{array}{cc} {\hat{x}}^{*}\left(k|k-1\right) & =L\;Z_{N}, \end{array}$$

(27) $$\begin{array}{cc} P^{*}\left(k|k-1\right) & =K_{N}Q_{N}K_{N}^{T}+LR_{N}L^{T}, \end{array}$$

(28) $$\begin{array}{cc} L & =J_{N}{\left[\begin{array}{cc} M_{1,1} & M_{1,2}\\ M_{1,2}^{T} & M_{2,2} \end{array}\right]}^{-1}\left[\begin{array}{c} {\bar{C}}_{N}^{T}\\ {\bar{G}}_{N}^{T} \end{array}\right]R_{N}^{-1}, \end{array}$$

(29) $$\begin{array}{cc} J_{N} & =[F^{N}\;\;F^{N-1}\;\;F^{N-2}\;\;\cdots \;\;F\;\;I], \end{array}$$

(30) $$\begin{array}{cc} M_{1,1} & ={\bar{C}}_{N}^{T}R_{N}^{-1}{\bar{C}}_{N},\\ M_{1,2} & ={\bar{C}}_{N}^{T}R_{N}^{-1}{\bar{G}}_{N},\\ M_{2,2} & ={\bar{G}}_{N}^{T}R_{N}^{-1}{\bar{G}}_{N}+Q_{N}^{-1}, \end{array}$$

(31) $$\begin{array}{cc} {\bar{C}}_{N} & =\left[\begin{array}{c} H\\ HF\\ HF^{2}\\ \vdots \\ HF^{N-1} \end{array}\right], \end{array}$$

(32) $$\begin{array}{cc} {\bar{G}}_{N} & =\left[\begin{array}{ccccc} 0 & 0 & \ldots & 0 & 0\\ HG & 0 & \ldots & 0 & 0\\ HFG & HG & \ldots & 0 & 0\\ \vdots & \vdots & \vdots & \vdots & \vdots \\ HF^{n-1}G & HF^{n-2}G & \ldots & HG & 0 \end{array}\right], \end{array}$$

(33) $$\begin{array}{cc} R_{N} & =\left[\mathrm{diag}(\overset{N}{\overset{︷}{R^{*}\left(k-N\right)\;R^{*}\left(k-N+1\right)\;\cdots \;R^{*}\left(k-1\right)}})\right], \end{array}$$

(34) $$\begin{array}{cc} Q_{N} & =\left[\mathrm{diag}(\overset{N}{\overset{︷}{Q^{*}\left(k-N\right)\;Q^{*}\left(k-N+1\right)\;\cdots \;Q{\left(k-1\right)}^{*}}})\right], \end{array}$$

(35) $$\begin{array}{cc} Z_{N} & ={\left[{\bar{z}}^{T}\left(k-N\right)\;\;{\bar{z}}^{T}\left(k-N+1\right)\;\;\cdots \;\;{\bar{z}}^{T}\left(k-1\right)\right]}^{T}, \end{array}$$

(36) $$\begin{array}{cc} K_{N} & =[F^{N-1}G\;\;F^{N-2}G\;\;\cdots \;\;\mathrm{FG}\;\;G]. \end{array}$$

Equations (26) and (35) indicate that the MVFF cannot handle clutters, and the most suitable measurement should be selected at each time step on the horizon [k−N,k−1]. The most suitable measurement can be selected using the nearest neighbor method as follows:

(37) $$\begin{array}{cc} \bar{z}\left(l\right) & =\underset{z_{i}\left(l\right)}{\arg\min}{\left(z_{i}\left(l\right)-\hat{z}\left(l\right)\right)}^{T}S_{i}^{-1}\left(l\right)\left(z_{i}\left(l\right)-\hat{z}\left(l\right)\right),\;\;\;\;i=1,\cdots ,m\left(l\right),\\ \hat{z}\left(l\right) & =H\hat{x}\left(l|l\right),\\ S_{i}\left(l\right) & =HP\left(l|l\right)H^{T}+R_{i}\left(l\right), \end{array}$$

where *l* is the time on the horizon [k−N,k−1].

Equations (33) and (34) indicate that the MVFF can handle time-varying noise covariances. In (33), $R^{*}\left(l\right)\;\;\left(l=k-N,\cdots ,k-1\right)$ can be obtained using $\bar{z}\left(l\right)$ and (6). To this end, $\bar{z}$ can be converted into a polar coordinate using the relation as follows:

(38) $$\begin{array}{cc} r_{m} & =\sqrt{x_{m}^{2}+y_{m}^{2}},\\ \theta_{m} & =tan^{-1}\left(\frac{y_{m}}{x_{m}}\right). \end{array}$$

In (34), $Q^{*}\left(l\right)\;\;\left(l=k-N,\cdots ,k-1\right)$ is determined by selecting one of the process noise covariances of the four models ($M_{1}$–$M_{4}$). The process noise covariance of the model producing the highest mode probability is selected as $Q^{*}\left(l\right)$.

After the outputs of the MVFF, ${\hat{x}}^{*}\left(k|k-1\right)$ and $P^{*}\left(k|k-1\right)$, are obtained, all the PDA filters in the main filter are reset using:

(39) $$\begin{array}{cc} {\hat{x}}^{j}\left(k|k\right) & ={\hat{x}}^{*}\left(k|k-1\right), \end{array}$$

(40) $$\begin{array}{cc} P^{j}\left(k|k\right) & =P^{*}\left(k|k-1\right),\;\;\left(j=1,2,3,4\right). \end{array}$$

The overall HIPFF algorithm is summarized in Algorithm 1.    
**Algorithm 1: **HIPFF
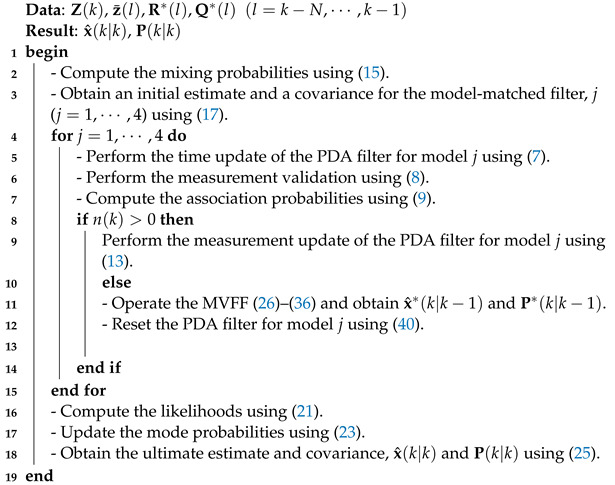


## 5. Simulation Results

In this section, the simulation results are presented to demonstrate the performance of the HIPFF. In the simulation, the 2D positions and velocities of a preceding vehicle were estimated using both the HIPFF and the IMMPDAF. The simulation scenario was as follows. Preceding and host vehicles were traveling at the same velocity on the highway. Subsequently, the preceding vehicle suddenly decelerated, and eventually stopped. The FCW system of the host vehicle estimated the position and velocity of the decelerating preceding vehicle. Various values of the initial velocities and relative distance of the vehicles were tested.

Radar clutters (false measurements) were generated independently, and were uniformly distributed in a square centered at the true measurement. The area of the square, $\bar{V}$, was computed using:

(41) $$\begin{array}{cc} \bar{V} & =N_{F}/\lambda ,\\ N_{F} & ={\left[10\pi \gamma |S\left(k\right)\right|}^{1/2}{\lambda +1]}^{-}, \end{array}$$

where $N_{F}$ is the number of false measurements and λ is the clutter density. In addition, the notation ${\left[\cdot \right]}^{-}$ corresponds to “rounded down to the nearest integer”, and S(k) was obtained using a standard Kalman filter under the assumption of an ideal (no clutters) environment [17].

Automotive radars can detect objects within a field of view (FOV). In the simulation, it was assumed that a Delphi Electronically Scanning Radar (ESR) was used for the FCW system. The Delphi ESR is an automotive frequency modulated continuous wave (FMCW) radar. The maximum detection range and FOV of the Delphi ESR in the long-range mode are 174 m and $20^{\circ }$, respectively. In addition, the maximum detection range and FOV in the mid-range mode are 60 m and $90^{\circ }$, respectively.

The sampling interval, target detection probability, gate probability, and gate threshold were set as T=0.1 s, $P_{D}=0.9$, $P_{G}=0.99$, and γ=9.21, respectively. The noise variances of the range/bearing measurements were set as $\sigma_{r}=0.25$ m and $\sigma_{theta}=1.5{\;}^{\circ }$ [9,10]. Trajectories of preceding vehicle were generated using the DWNA model (1) with the standard deviation of process noise, $\sigma_{w}=0.08$. For the four motion models, $M_{1}$–$M_{4}$, used for IMM estimation, the standard deviations were set to 1, 1, 10, and 0.1, respectively, and the horizon size of the MVFF was set as N=4.

The performance of the estimation algorithms was evaluated using the root-mean-square position error (RMSPE) and the root-mean-square velocity error (RMSVE), which can be calculated using:

(42) $$\begin{array}{cc} \mathrm{RMSPE}(k) & =\frac{1}{N_{MC}}\sum_{i=1}^{N_{MC}}\sqrt{{\left(x\left(k\right)-{\hat{x}}^{i}\left(k\right)\right)}^{2}+{\left(y\left(k\right)-{\hat{y}}^{i}\left(k\right)\right)}^{2}}, \end{array}$$

(43) $$\begin{array}{cc} \mathrm{RMSVE}(k) & =\frac{1}{N_{MC}}\sum_{i=1}^{N_{MC}}\sqrt{{\left(v_{x}\left(k\right)-{\hat{v}}_{x}^{i}\left(k\right)\right)}^{2}+{\left(v_{y}\left(k\right)-{\hat{v}}_{y}^{i}\left(k\right)\right)}^{2}}, \end{array}$$

where $N_{MC}$ is the number of Monte Carlo (MC) simulations; (x(k),y(k)) and $\left(v_{x}\left(k\right),v_{y}\left(k\right)\right)$ are the true 2D positions and velocities, respectively; and $\left({\dot{x}}^{i}\left(k\right),{\dot{y}}^{i}\left(k\right)\right)$ and $\left({\hat{v}}_{x}^{i}\left(k\right),{\hat{v}}_{y}^{i}\left(k\right)\right)$ are the estimated 2D positions and velocities obtained in the *i*-th MC run, respectively. The RMSPE and RMSVE values were computed using 1000 MC runs. In addition, the percentage of lost track among the 1000 tracks was computed. A lost track was determined if the mode probability of $M_{1}$ exhibited the highest value for more than 20% of the simulation time.

**Figure 2:**
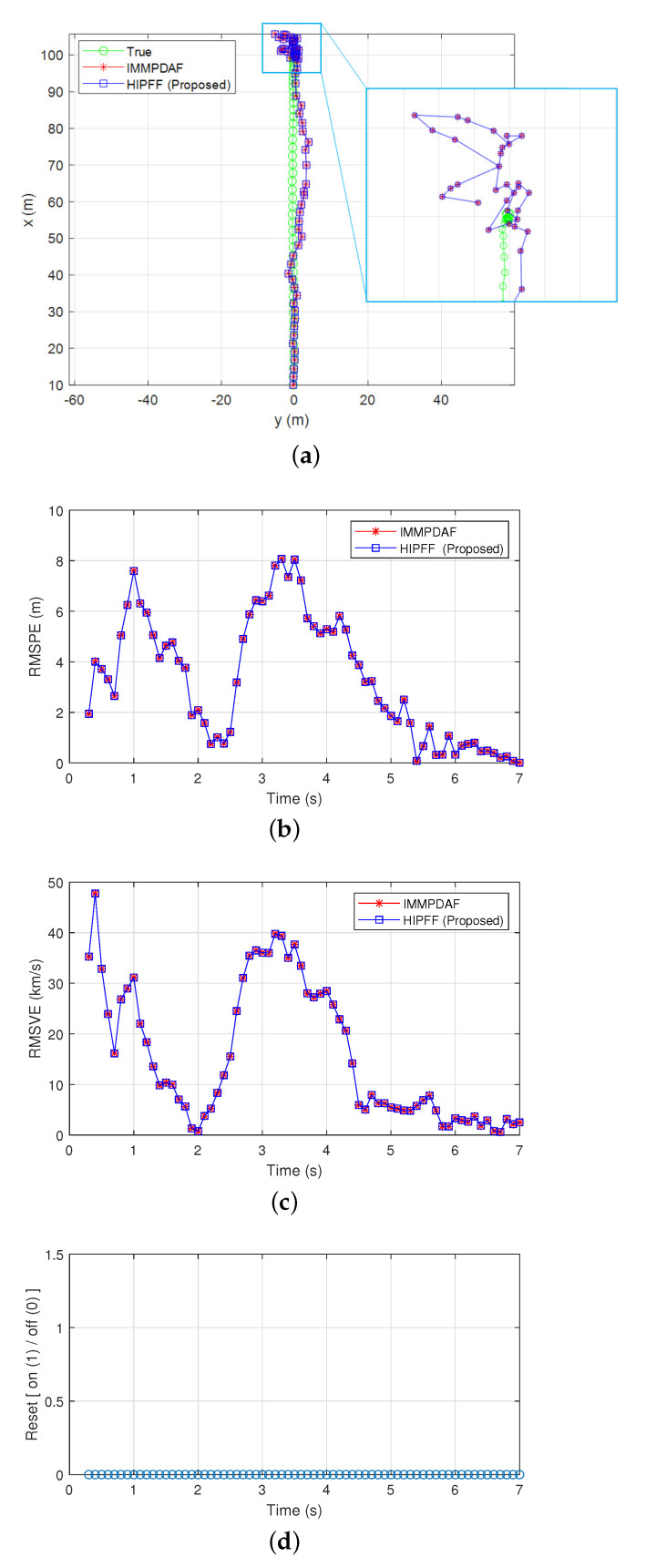
Simulation results of a successful track case: (**a**) true and estimated positions; (**b**) root-mean-square position error (RMSPE); (**c**) root-mean-square velocity error (RMSVE); and (**d**) reset timing.

**Figure 3:**
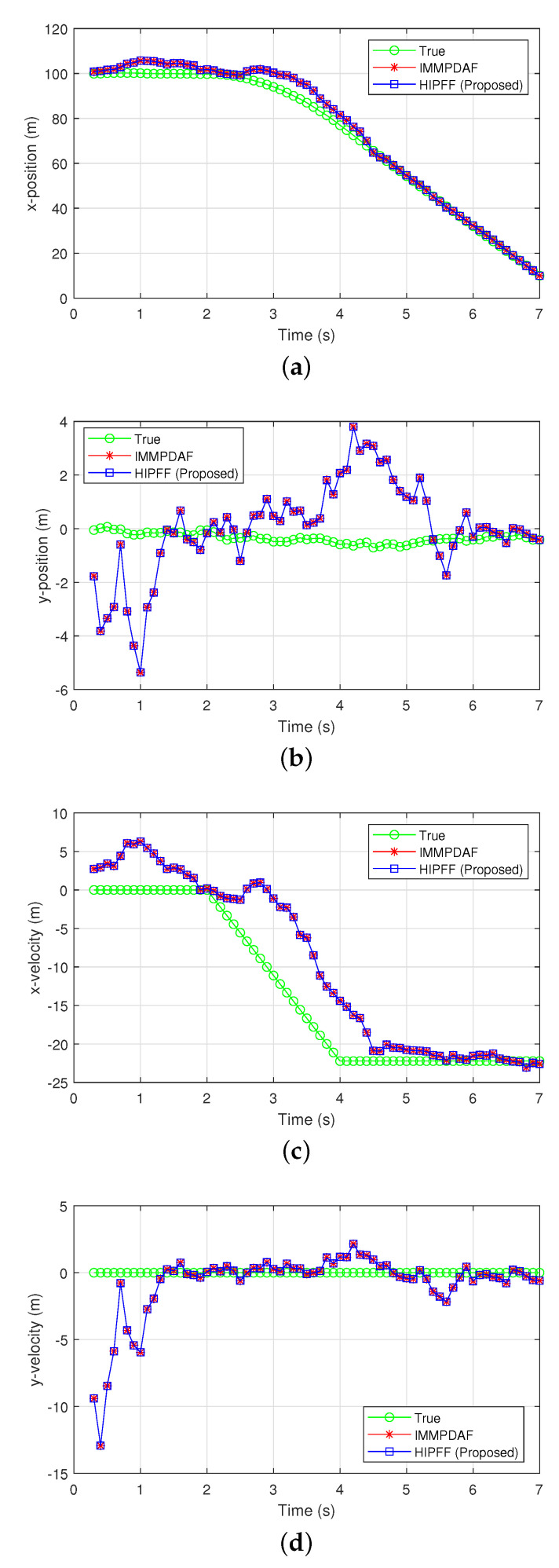
Estimation results of a successful track case: (**a**) x-position; (**b**) y-position; (**c**) x-velocity; and (**d**) y-velocity.

Figure 2 and Figure 3 show the simulation results of a successful track case when the initial distance between the preceding and host vehicles along the x-axis was $d_{0,x}$ = 100 m, and the initial velocities along the x-axis was $v_{0,x}=80$ km/h. The image revealed that the IMMPDAF did not diverge and successfully tracked the preceding vehicle under this condition. The preceding vehicle traveled at a velocity of 80 km/h for 2 s, after which it rapidly decelerated for 2 s and eventually stopped. The IMMPDAF did not always diverges and sometimes exhibited a successful track. However, under rapid deceleration, the IMMPDAF diverged often. If failures of IMMPDAF did not occur, the assisting MVFF did not operate. Subsequently, the HIPFF operated like a pure IMMPDAF and exhibited the same results as the IMMPDAF, as shown in Figure 2a–c. Figure 2d shows that a reset by the assisting MVFF did not occur. In addition, Figure 3a–d show the true/estimated positions and velocities as a function of time.

**Figure 4:**
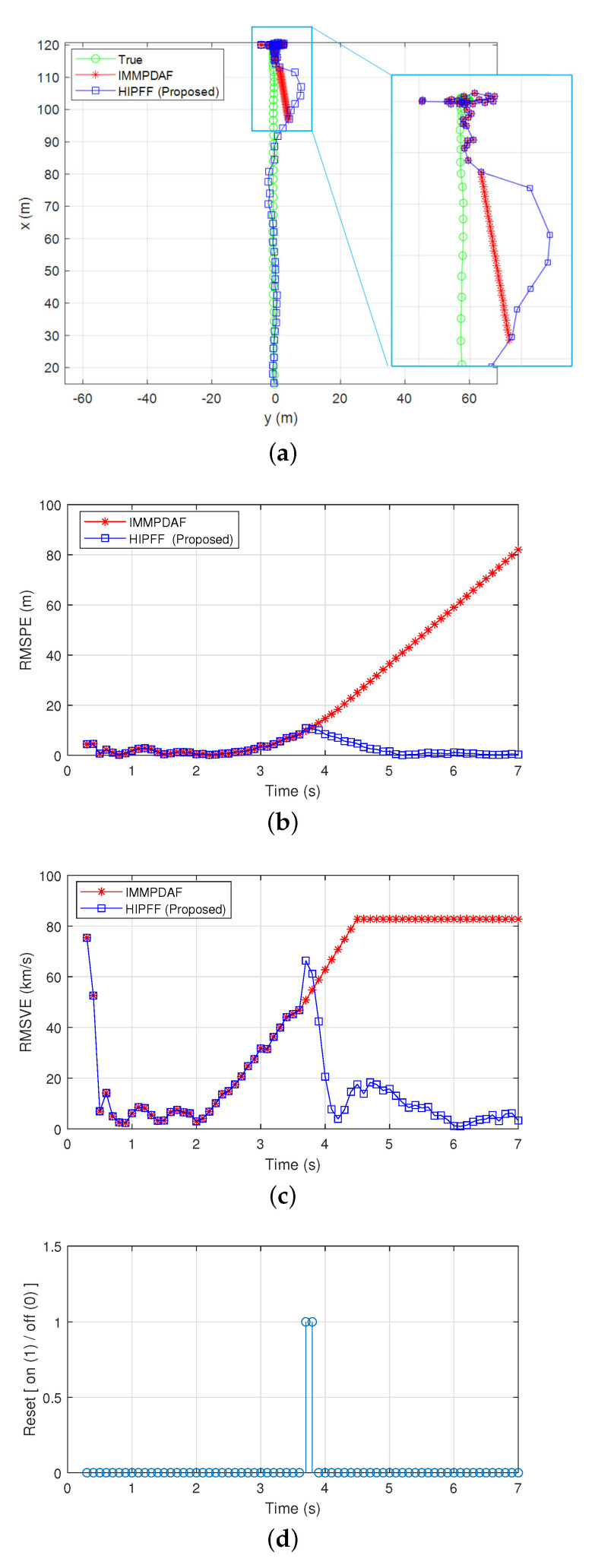
Simulation results in case of divergent interacting multiple model probabilistic data association filter (IMMPDAF): (**a**) true and estimated positions; (**b**) RMSPE; (**c**) RMSVE; and (**d**) reset timing.

**Figure 5:**
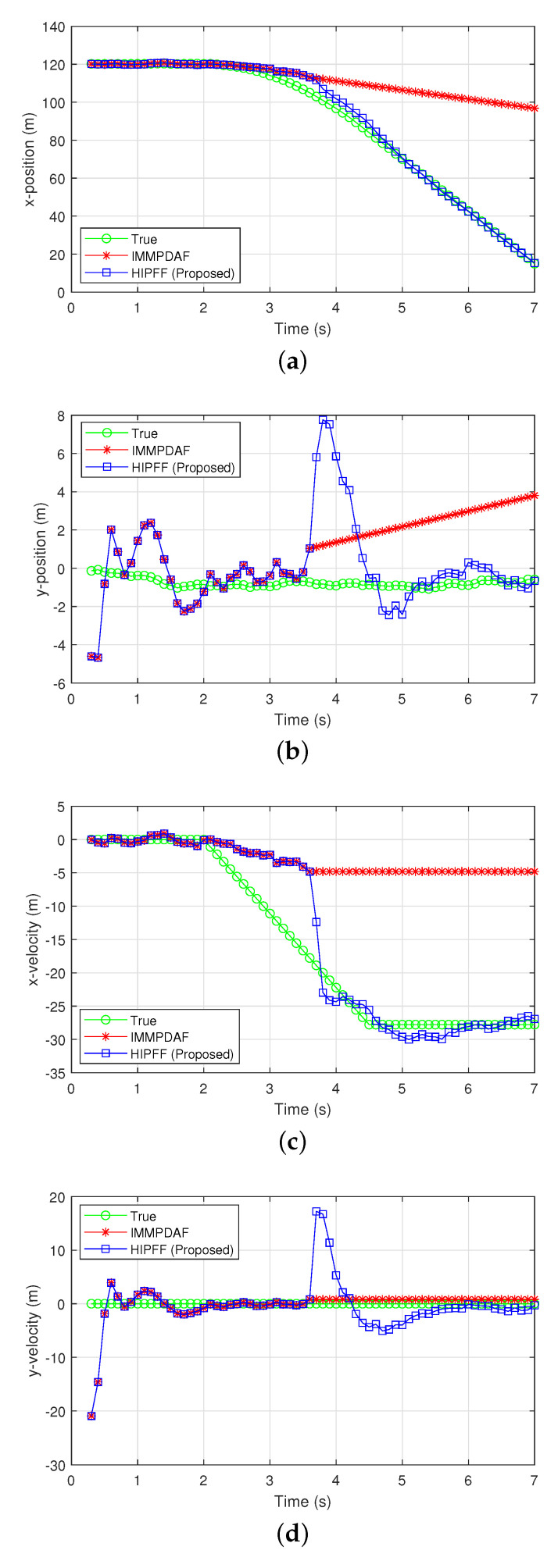
Estimation results of a successful track case: (**a**) x-position; (**b**) y-position; (**c**) x-velocity; and (**d**) y-velocity.

Figure 4 and Figure 5 show the simulation results of a divergent track case, where the IMMPDAF lost the preceding vehicle and the RMSPE of the IMMPDAF diverged. In this simulation, the initial distance between the preceding and host vehicles was $d_{0,x}=120$ m, and the initial velocity of the vehicles was $v_{0,x}=100$ km/h. The preceding vehicle traveled at a velocity of 100 km/h for 2 s, and rapidly decelerated for 2.5 s and eventually stopped. In Figure 4, the HIPFF did not diverge and successfully tracked the preceding vehicle. The RMSPE and RMSVE values of the HIPFF increased initially, after which it decreased owing to the reset by the MVFF (Figure 4b,c). Figure 4d shows that the resets occurred three times. Figure 5a–d show the true/estimated positions and velocities as a function of time.

Table 1, Table 2 and Table 3 show the MC simulation results under two different clutter levels: heavy and medium. λ=5×10${}^{-2}$ and λ=1×10${}^{-2}$ were used for the heavy and medium clutter conditions, respectively. In addition, three different initial distance and velocities were tested. Table 1, Table 2 and Table 3 show the time-averaged RMSPE and RMSVE values, and the percentages of the divergent tracks. The proposed HIPFF exhibited a higher accuracy than IMMPDAF in terms of the averaged RMSPE and RMSVE values. Moreover, the HIPFF was significantly more reliable than the IMMPDAF in terms of the percentage of divergent tracks. This demonstrates that the HIPFF is significantly more accurate and reliable than the conventional IMMPDAF under severe conditions.

## 6. Conclusions

This study proposed a novel state estimation algorithm, namely hybrid IMM-PDA/FIR filter (HIPFF), for forward collision warning (FCW) systems based on automotive radars. The proposed algorithm utilized interacting multiple model probabilistic data association filter (IMMPDAF) as the main filter, and a finite impulse response (FIR) filter (i.e., minimum variance FIR filter (MVFF)) for recovering the main filter from failures. Under a rapidly decelerating preceding vehicle condition, the simulation results revealed that HIPFF exhibited significantly accurate and reliable estimation of position/velocity, compared to that of the pure IMMPDAF. Therefore, the HIPFF algorithm is expected to exhibit improved performance for automotive radar-based FCW systems.

## Figures and Tables

**Table 1:** Monte Carlo (MC) simulation results under the condition of $v_{x,0}=120$ km/h, $d_{x,0}=140$ m.

Clutter Level	Heavy		Medium	
	IMMPDAF	HIPFF	IMMPDAF	HIPFF
Averaged RMSPE (m)	24.25	4.98	21.89	4.59
Averaged RMSVE (km/h)	53.98	33.94	49.39	47.23
Percentage of divergent track	95.4	0	91.7	2.7

**Table 2:** MC simulation results under condition of $v_{x,0}=100$ km/h, $d_{x,0}=120$ m.

Clutter Level	Heavy		Medium	
	IMMPDAF	HIPFF	IMMPDAF	HIPFF
Averaged RMSPE (m)	22.21	4.29	19.29	3.7
Averaged RMSVE (km/h)	47.06	30.93	41.68	38.1
Percentage of divergent track	92.4	0	87.6	0.6

**Table 3:** MC simulation results under condition of $v_{x,0}=80$ km/h, $d_{x,0}=100$ m.

Clutter Level	Heavy		Medium	
	IMMPDAF	HIPFF	IMMPDAF	HIPFF
Averaged RMSPE (m)	19.39	3.63	14.76	2.74
Averaged RMSVE (km/h)	39.15	28.2	31.37	29.49
Percentage of divergent track	88.9	0	75	1.7

## Abbreviations

The following abbreviations are used in this manuscript:

ADAS	Advanced Driver-Assistance Systems
FCW	Forward Collision Warning
GPS	Global Positioning System
TTC	Time-To-Collision
FIR	Finite Impulse Response
PDA	Probabilistic Data Association
IMM	Interacting Multiple Model
IMMPDAF	Interacting Multiple Model Probabilistic Data Association Filter
HIPFF	Hybrid IMM-PDA/FIR Filter
2D	Two-Dimensional
KF	Kalman Filter
DWNA	Discrete White Noise Acceleration
MVFF	Minimum Variance FIR Filter
FOV	Field of View
ESR	Electronically Scanning Radar
RMSPE	Root-Mean-Square Position Error
RMSVE	Root-Mean-Square Velocity Error
